# Insanity and Intemperance

**Published:** 1863

**Authors:** Andrew McFarland


### INSANITY AND INTEMPERANCE.

BY ANDREW McFARLAND, M.D.

From the "American Journal of Insanity."

Among the problems of psychological science which remain to be solved, is, such a discrimination between the manifestations of mental disease and some of the effects of the habitual use of diffusible stimulants as will render reasonably clear the administration of justice in criminal courts. It is not merely with the broad resemblances between insanity and drunkenness that we have to deal, in some of the cases which occur; not the question how far a fit of intoxication renders the individual irresponsible for what he does; but we sometimes have the two states conjoined in the same individual, each with its liabilities and immunities, making a skein of commingled guilt and irresponsibility, which science must disentangle. We must sometimes throw so much light on the tissue of testimony held up before us, that amid all its intertwisting. what is the indelible coloring of disease, and what the transient stain of a vicious habit, shall at once appear. The task is a difficult one, requiring a nice analysis of their differences, and such a bold separation of them that justice may plainly see where to strike.

In two instances, within the last year, the subject of insanity in connection with the excessive use of stimulants, has presented itself in the courts of Illinois, where the two conditions could be viewed in their relation to each other.

The first case, *Keenan vs. Van Horn*, had little of interest, except for the decision rendered, which goes somewhat to open an enlightened procedure in such cases. In this case, suit was brought by the complainant, Margaret Keenan, to recover possession of certain property conveyed by her husband during his life to Van Horn, while incapable of so doing, by reason of mental disease.

The deceased was long in the habitual and excessive use of ardent spirits, resulting finally, as was claimed, in permanent mental disease. The testimony, which was very voluminous, proved that for fifteen or twenty years, he had been a common drunkard, that his propensity for such indulgence grew more inveterate, terminating at last in his death from dropsy and general decline. Toward the last of his life he had abandoned his family and taken up his residence with Van Horn, to whom he conveyed his homestead and other effects without adequate consideration.

The allegation of his incompetency rested chiefly on certain distinct and strongly marked peculiarities, which always attended him when under the influence of liquor. At such time he fancied himself a military commander, styling himself "Capt. Rock," and would spend many successive days and nights in giving the word of command to imaginary companies of soldiers, whom he extemporized out of sticks of wood, stumps of trees, &c., and that his fits always took that form and no other. At such times it also appeared that he had no adequate idea of the value of money, but spent it lavishly in buying articles for which he had no use, or which he gave away to persons in whom he had no interest. Testimony as to his condition during the intervals between his fits of drinking was somewhat conflicting, though the weight of it seemed to be that, with the exception of his faculties being somewhat blunted, there was nothing very different in him from other men.

In this case it was held that the unvarying recurrence of the mind of the deceased to certain fixed and unchanging delusions, was evidence, notwithstanding the occasion of such delusions may have been induced by indulgence in liquor, that there was radical mental impairment, that there was a difference in his peculiarities from the ordinary phenomena of the drunken fit, the aberrations of the latter being more general or diffuse, and not commonly attended with such special delusions as was shown always to exist in this case. The liquor was claimed to act, in this instance, upon certain always present, though latent, diseased mental traits,—something like the effect of a varnish upon the grain of a wood,—bringing into view what was before invisible, though none the less present.

Judgment was rendered for the plaintiff in this case, from which an appeal was made to the Supreme Court, which, however, sustained the decision.

It must not be understood that those general but always appearing traits which some persons exhibit when inebriated, are included in this view. Some men, for instance are always dignified, some quarrelsome, and some amorous when in their cups, and some indeed, like Mr. Snevellicci, pass through all those stages in the course of a single bout. A difference will be recognized between this exhibition of some general trait, and that taking up of a special idea, which was held, in this instance, to be indicative of fundamental impairment of the intellect.

This case is cited rather by way of introduction to another of much greater importance, in which this distinction is more clear, and becomes more necessary.

William Hopp was tried for the murder of his wife before the Circuit Court of Cook County, in December last, Judge Mannierre presiding. The trial was protracted, excited deep interest, and has points well worthy professional attention.

Hopp is an Englishman, who came to this country with a younger brother, and settled near the head of Lake Champlain, in Vermont, perhaps thirty years since. Testimony of importance, in regard to the insanity of his mother and his aunt, was ruled out of the proceedings, as technically inadmissable. After residing some time in Vermont, both brothers moved to Illinois, and settled some twenty miles from Chicago. It was proved by the prosecution, by way of derogation of the character of Hopp, that while living in Vermont, he was engaged in smuggling goods across the Canada border. But all testimony in regard to him, since residing in Illinois, showed him strictly upright in every business transaction, and somewhat punctilious in matters of honor and veracity. By great industry and thrift, he acquired a handsome property, and was living, at the time of the homicide, in a style much above the average of his neighbors. It may be mentioned that Hopp had always used ardent spirits freely, though not regarded as an intemperate man. Some years after coming into the State, the younger brother became incontestably insane, and still remains so, though residing with, and cared for by his brother.

Twelve years ago William Hopp, while repairing a bridge, was exposed for several days in succession to a thorough wetting, and an obstinate dumb ague was the consequence. At this distance of time it is impossible to get at the exact state of his mind during this illness. But it appears that, while still suffering under its effects, he had a trifling difficulty with one of his neighbors, whose horse had died while in his (Hopp's) hands, though in no way made diseased by any labor or ill-usage. After some dispute, an arbitration followed, in which it was decided that Hopp should pay half the value of the animal. He appeared unusually disturbed by this transaction; his mind seemed to dwell upon it to the exclusion of almost everything else. He fancied it not less an act of injustice than an imputation upon his personal honor. What increased his vexation was an idea that his wife was indifferent to his interests in the transaction; and this impression finally changed into a conviction that she was in complicity with the arbiters who had made the decision.

From this period commenced a course of personal abuse, occurring in paroxysms, in which he charged her with unchaste conduct, at first with these particular parties, and at length with a prostitution almost indiscriminate. It may be mentioned that no woman could exist in whom such accusations could be more unfounded. These periods of abuse were strictly periodical, leaving him, during the interval, affectionate and considerate as other men. But they increased in frequency and length, sometimes continued with hardly any cessation for two or three successive days and nights. This abuse commenced, at first, in the form of remonstrances against her unchaste conduct. Then it took the form of most profane and obscene epithets, coupled at last with extreme personal violence. He never applied any epithet to her except such as denoted unchastity. In the presence of others, during all the early part of this period of ten years, he treated her with due consideration. Only his children were witnesses to it, by overhearing him after he and his wife had retired. But at last the presence of his children, and finally of strangers, made little difference. These paroxysms were attended by the consumption of large quantities of liquor, and the degree of his abuse of his wife was measured, in the estimation of his neighbors and children, solely by the depth of his potations. Sometimes, exhausted by this protracted persecution, she would leave him, threatening not to return. No sooner would she be out of his sight than he seemed a changed man. He would abstain wholly from drink, become penitent and full of self-reproaches, write beseeching letters imploring her return, and even take oaths before a magistrate to abstain forever from liquor, upon which he charged all his conduct. But as soon as she comes again in his sight, the same abuse is renewed, even before they had reached the house from the cars in which she had returned. During these years, all the testimony showed that as a father and a neighbor he was exemplary. He was a man reserved in the extreme in imparting his confidences, and never, except in occasional obscure hints, disclosed his impressions regarding his wife's unchastity. He clearly did so, but in rare instances, and only to those in whom he had most implicit confidence.

His wife seemed the only person who had any idea of the true cause of his singular conduct. That she had such idea, appears from her frequently advising him to take calomel and other medicine.

In the month of June, 1862, he returned in the evening from a neighboring village intoxicated, but not as much so as on many former occasions. He commenced his abuse in the usual terms, to which she made little reply, when, as she was seeking to evade him, he struck her, while passing, with a knife, which inflicted a wound in the abdomen, of which she died about twelve hours afterwards. On the assembling of the neighbors, Hopp appeared perfectly calm and unconcerned. He calls them to witness his present sobriety, tells them the act was a deliberate one, and contemplated for the past ten years.

Just previous to his trial, the writer of this article visited him in the jail, the prisoner having no idea whatever of the person, or the object of the visit. The prisoner is about fifty-eight years of age, rather above the common height, and of fair intelligence for one of his class. His honesty and sincerity are unquestionable, and his statements in regard to the tragedy and the ideas antecedent to it, bear the stamp of perfect ingenousness. He went into a lengthened narrative of his troubles, commencing with the arbitration in reference to the horse. The proofs of his wife's infidelity, which he circumstantially narrates, are the merest "stuff of which dreams are made." As evidences of the trifles on which the insane base their delusions they possess a certain degree of interest.

On one occasion, for illustration, when a son was born to Hopp, certain acquaintances, and among them one of the arbiters in the horse case, assembled in honor of the event. A toast was drunk complimentary to Hopp, especially in relation to his ability to beget children. This he regarded as clear proof that the proposer of the toast thereby acknowledged the guilt of which Hopp had previously suspected him. A remark made afterward by the same individual, that "women were good creatures," was conceived to have the same import. As was before remarked, his conviction of his wife's infidelity so widened, during the last of her life, as to include most persons who even approached his dwelling. An individual who had called to purchase some onions, in Hopp's absence, was regarded by him as the father of one of his children, and, on calling at the house some months afterwards, the child was brought out by Hopp and introduced, by way of test, as "the little onion boy." In narrating the circumstance of this introduction, Hopp concludes with the remark that if the individual thus accused had "spoke volumes of confession, it would not have been equal to the look of guilt which that introduction created."

No one at all acquainted with the manifestations of mental disease will fail to recognise a state of mind of which such ideas as the above form a texture, as insanity of the most unequivocal type. Yet, never was there prisoner arraigned at the bar more completely shorn of every vestige of sympathy, or who stood entirely alone in his extremity. Fully justifying himself in what he had done, he seemed to conceive that all he had to do was to make statements, of which the narration is a specimen, to convince all others of his innocence. He had no idea, before the trial, of the plea which was to be set up for him. No testimony against him was so unrelenting as that of his adult daughters', who urged the prosecution with a vindictiveness as great as if the blood in their veins was drawn from the most opposite sources.

An attempt was made by the expert testimony, to show that the violent conduct of Hopp for ten years before the homicide was purely the result of a delusion; that, dating from about the time of the arbitration, he was an insane man; that his insanity was evidently hereditary, though induced by the illness of which mention has been made; that his delusion having assumed the form it did, was merely accidental, and that it was no more strange in him to have accused an innocent woman of promiscuous intercourse with chance-comers to the house, than are the innumerable other forms which the mysterious disease of insanity perpetually puts on. The cool-blooded atrocity of the act of homicide, and the indifference and self-justification of its perpetrator were shown to be strictly in accordance with the nature of mental disease, as it existed in the prisoner; that, believing her continuance in guilt was more to be deplored than her death, he become her executioner, and, by the perverted operation of his reasoning powers, he expected justification for the act he was committing.

It was urged that the habit of drinking was not the sole cause of the homicide, as contended for by the prosecution, but a mere incident, having, quite likely, little or nothing to do with the disease; that, had his conduct proceeded from indulgence in liquor alone, he would have shown quarrelsome and violent dispositions toward others as well as his unoffending wife; that the special terms which he invariably used toward her were significant of the singleness of the idea under which he existed; that, had the fatal blow been struck as the mere impulse of a drunken fit, the consequences of what he had done would have so shocked him as to have driven the fumes of liquor from his brain at once, and produce a paroxysm of remorse, while his whole demeanor, from that time till the inquest, was that of indifference and self-justification.

It was further shown that the change which took place in the mind of the prisoner when his wife was absent, was one of the ordinary phenomena present in all cases of delusion, and in accordance with the law of mental disease; that where a delusion appends to another person, it disappears for the time being when the person is out of sight, and the fact of delusion is proved by the disappearance or the idea with the disappearance of the person to whom it relates. The clearly defined beginning of his altered conduct towards his wife, is also cited as one of the proofs that his conduct was the result of disease, and not of intemperate indulgence. It does not appear in any testimony, that his treatment of his wife was unkind, till the time of the arbitration before alluded to; and yet he was decidedly intemperate many years before that transaction.

Much stress was laid, in the prosecution, upon the oft-repeated declaration, "that he never abused his wife except when he was in liquor." This may all be true, and yet, if accepted as a bald statement, allows a fatal prejudice to enter into the case. It needs no wide experience to show how commonly the approach of a fit of paroxysmal insanity is signalled by an inordinate thirst for artificial stimulants, and how certainly the subject of that form of disease will avail himself of them if within his reach. William Hopp, with ample means, was always prepared thus to feed a natural excitement with an artificial one, and he always did so, is merely proof that the coming on of the paroxysm was invariably attended with certain irresistable cravings. So far from it being a fact, that the homicide was merely the result of this indulgence, the theory is by no means untenable that the habit of drinking actually postponed the fatal tragedy, upon the well-known principle in mental philosophy, that the purposes of the will are dissipated and made ineffective under the diffusive tendencies of alcoholic stimulants.

In all human probability, in this instance, the fixed purpose of the lunatic was sometimes lost sight of in the windy brawl of the drunkard.

The charge of Judge Manierre is worthy of being quoted at considerable length. Viewed in the light of an attempt to make a difficult subject understood by a jury of plain men, it is certainly a success. Though there are many ideas in it at which exception would be taken, it has certainly the merit of great lucidity, and stands in striking contrast with the "muddle" uttered from the bench in the case of Real, quoted in the last Journal of Insanity. It may be remarked, that some of the former expositions of the law of insanity promulgated by Judge Manierre, especially in the Green case, tried in Chicago some eight years ago, entitle his views to high consideration, and will be regarded, even by those who differ in some of them, with sincere respect. It should be explained that during the trial, the usual passage-at-arms took place between the counsel for the prosecution and a witness expert, on the subject of *moral insanity*—wholly foreign to the points of the case, and intended for mere effect. The somewhat lengthened discussion of this subject may have led the Court to the frequent allusions to it, which appear in the remarks from the bench:—

REMARKS IN GENERAL.

"A crime," says Judge Manierre, "is defined as a violation of a public law, in the commission of which there shall be an union of act and intention. Intention is manifested by the circumstances surrounding the act, indicating its motive or object, and the sound mind and discretion of the accused. A person shall be considered of sound mind who is neither an idiot nor lunatic, nor affected with insanity, who has a knowledge and consciousness of the distinction between good and evil. In this case, the homicide is admitted, but the accused alleges that at the time of the commission of the act his mind was so affected with insanity, that his moral sense and will were subjected by it, and he was oblivious to the moral quality of the act. The law presumes the sanity of every person charged with a criminal act, and that such act is the result of volition influenced by motives acting upon the mind. Hence the burden of overcoming this presumption rests upon the accused; but when insanity is satisfactorily shown, it is the duty of the jury to acquit, as in such case there is an absence of intention which is essential to a criminal act.

"Insanity is, generally, classified into moral and intellectual, and is either general or partial. Moral insanity consists in a disorder of the moral affections and propensities without any symptom of delusion or error impressed upon the understanding. Intellectual insanity is a disorder of the intellect, and is characterized by delusion or hallucination of mind, manifesting itself either in the belief of things naturally impossible, or of facts so implorable when considered in connection with the evidence upon which the belief is formed that no person in his senses could believe them. But these general definitions do not afford to the unprofessional mind a sufficiently clear and comprehensive idea of insanity thus classified and defined, to enable it to apprehend those distinctions of science and law which are necessary to the formation of a judgment in this case. And it is due to the accused when such tremendous issues are involved as here, that those distinctions should be marked and defined with the utmost care and exactness by the court.

"The mind, in its more general sense, includes not only the powers of the understanding, as perception, reflection, imagination, memory, will, and judgment, but also the moral sense or conscience, and the disposition, propensities, affections, and passions. The passions, inclinations, and propensities indicate the state or impulses of the mind, and constitute what are termed the moral powers, as contradistinguished from the intellectual. The action of the intellect can only manifest itself to the observation of others through the action or conduct of the individual. All actions proceed from the passions or from motives acting upon the mind and influencing the judgment and will. We judge of the character of a man by his conduct, and as that is regulated by just or evil impulses, we determine the moral constitution of his mind. When, therefore, we speak of the moral powers, we are understood to refer to the propensities, disposition, or temper of the mind; whilst on the other hand, when we speak of the intellectual powers, we refer to the faculties of judgment, will, and conscience.

"Thus constituted, man is regarded by law as a free moral agent, endowed with the power of volition or choice among different motives presented to the mind, and of determining whether his conduct shall be good or evil. It also assumes that every man has the power of determining whether an act is right or wrong, and that upon the existence of this moral sense and freedom of will that all law, human and divine, bases its authority and its sanctions. If a man was obliged to do exactly what he does—if, in other words, he had no liberty of choice between good and evil, and his judgment and will must yield to any motive, impulse or passion acting it—then the whole system of criminal jurisprudence is founded upon an error, both fundamental and ineradicable. Free and moral agency implies the entire subordination of the passions and propensities, or moral powers, to the will, and the power of the will to control them, and assumes that all the outward acts and conduct are directed or suffered by the will, and hence that they are voluntary. On this principle, society, in all its relations, reposes. It is applied without regard to the moral training of the individual in youth, or to irritability of disposition arising from disease, or from temper, or passions habitually indulged. However perverted the moral sense or strong and uncontrollable the passions, the individual is, nevertheless, presumed to be possessed of a sense of right and wrong, and the power to control the will and to act from choice, and this presumption cannot be rebutted by any evidence which falls short of proof of insanity.

OF INTELLECTUAL INSANITY.

"We may now perceive more clearly what is meant by insanity, both mental and moral. And first of intellectual insanity:—The characteristic mark of this affection or disorder of the intellect, is delusion or hallucination, and is either general or partial. In general mania, the hallucination extends to all kinds of objects and subjects, and generally manifests itself in frenzy or raving madness. In monomania or partial insanity, the hallucination is confined to a single object or a small number of objects. This is the species with which we have here to do.

"Its true legal characteristic is delusive, or that state of the mind which is indicated by a belief in something, in itself, morally impossible. As, that trees walk, statues nod; or in the belief of a state of facts, in their nature, morally possible, but of the existence of which there is an entire absence of all reasonable grounds of belief. It also sometimes manifests itself in a belief of a direct revelation and of a controlling and irresistable sense of obligation to obey the revealed will.

"This state of the intellect indicates the existence of a disease, which, in its effects, subjects the will, judgment, and conscience to the imagination with respect to the subject of the insane belief. *The influence of such belief or delusion over the mind is much greater than the power of any conviction or belief in the mind of a sane person, and directs and controls the will,*

*judgment, and moral sense with inconceivably greater force.* The individual thus affected may be able, in most respects, to reason correctly on any subject beyond the range of his hallucination, and be not unfitted for the intelligent care and oversight of his business. Nor is the power of judgment and reasoning disturbed in any perceptible degree, even with respect to the subject of the delusion, as his conduct and reasoning are as logical and rational with respect to it as if the facts constituting the delusion were real and not imaginary.

"The law, as well as medical science, recognises all these forms of mental insanity, and has certain established principles applicable to the subject. For obvious reasons, a higher degree of insanity must be shown to absolve a party from the consequences of criminal acts than to discharge him from the obligation of his contracts. A man is not to be excused from responsibility if he has capacity and reason sufficient to distinguish between right and wrong as to the particular act he is then doing, a knowledge and a consciousness that the act is wrong and criminal. But in these cases it is not deemed sufficient that the individual has a general knowledge that the act is wrong in its nature, because this general knowledge may well consist with delusion as to the moral quality of the act, when considered in reference to the person and the circumstances believed to exist, and which in themselves constitute the delusion or insanity. There may be insane delusion with respect to one's moral duty under such circumstances, as well as in the belief, which is the primary evidence of unsoundness of mind. From whatever cause the power of the will or conscience may be subjected or perverted by an insane affection, self-agency ceases, and acts done under the influence thereof are neither criminal nor punishable, because they are not considered voluntary. For this reason the law will excuse homicide on the ground of partial insanity in the following cases:—

"*First.*—When the accused takes life under circumstances in which the act would be excusable if the facts constituting the delusion had an actual existence, and were not mere hallucinations, as in defence of life or habitation.

"*Second.*—When the act is done under a delusive belief of a Divine command and overruling necessity, or under a controlling sense of moral duty, which deludes and misleads the understanding and conscience with respect to the moral quality of the act.

"*Third.*—When the delusion consists in the belief that a wrong has been done to the accused in a manner, which, if true as believed, would not excuse homicide, but he is at the time of the commission of the act, so affected by the disease as to be incapacitated from knowing that he is doing wrong, and is unconscious of wrong. But where such knowledge and consciousness exist, the accused cannot be acquitted on this ground, as the act will be treated as one of revenge."

Certainly, the above will be accepted as very fair elucidation of the principles of mental disease, as they apply to the general order of cases. The nature, and especially the force of a delusion, (expressed in the passage italicized in the re-print,) will be regarded as very well conceived, though few will agree in a subsequent statement that "a higher degree of insanity must be shown to absolve a party from the consequences of criminal acts, than to discharge him from the obligation of his contracts."

The popular idea of "moral insanity" is well expressed in the following observations. All is certainly conceded which the most strenuous advocate of that distinction of a disease can desire. The industrious distribution of the "Huntingdon trial"—that scientific *morceau* being the sum total of the literature of insanity which many a Western law library can boast—has given those who oppose the plea of insanity, indiscriminately, some excellent matter for redicule. As before hinted, those who now sustain the plea of insanity as witnesses, have to meet the broad burlesque on the subject which this book virtually amounts to. A proposition was actually made in the Hopp trial to quote its medical opinions as the sanctioned views of "the doctors!"

OF MORAL INSANITY.

"As defined by those medical writers who treat this disease, it consists in the existence of some of the natural inclinations, dispositions, or propensities, in such violence that it is impossible not to yield to them. It is attended with no delusion or disorder of the mental faculties in any notable degree, and the mind is conscious of right and wrong while under its influence. And yet, notwithstanding this consciousness, the mere violence of the inclination to commit the act is so great as to overthrow all the power of resistance which the mind may be able to oppose to it. Under its influence the individual ceases to be a moral agent. When manifesting itself in the homicidal form, the inclination and desire to kill, is often indiscriminate in its violence, sometimes directing itself against the life of persons indifferent to the sufferer as well as against objects of affection and friendship, and it is impossible for him to restrain the uncontrollable fierceness of the impulse or desire. The act is never influenced by revenge or any of the passions or a desire to gain temporal advantages from the homicide. It is said to overcome the power of self-control, and to act without motive of any kind, and frequently without premeditation, and consists in the mere violence of the propensity or disposition by which the will is overcome.

"Most certainly, if this form of insanity has any existence, the doctrine of free agency can have no application to one affected with it. It is at least of exceedingly rare occurrence, and its manifestations, as it has been observed, bear a striking resemblance to crimes. Nevertheless, it is recognized by the medical profession, though it has been rejected by the English courts of justice as apocryphal. Yet it has been adopted by some courts of very high authority in this country, and what is of more consequence to us, it is implicitly recognized by the Supreme Court of this State in the case of Fisher. It is true it was not adopted in that case upon solemn consideration. Yet it must be regarded as the law of this case. But in saying this, it is my duty to add that it was regarded as so perilous in the administration of justice by the Court which first promulgated it as a principle of legal science, as to induce the observation, that this mania is dangerous in its relations, and can be recognized only in the plainest cases. It ought to be shown to have been habitual, or at least to have evinced itself in more than a single instance, or from its circumstances to bear unmistakable marks of instinctive and uncontrollable impulse. "Where this affection is alleged," says Dr. Ray, whose authority is one of the chief supports of this opinion, "in excuse for crime, it must be proved, first, that it was really present; second, that it had arrived at that stage in which its impulses are irresistable; thirdly, that it should be the exclusive cause of the criminal act.

"Governed by these rules there can be but little difficulty in determining the presence or absence of this disorder when it exists, and is really the cause of the criminal act, as it may be said that there can be no reliable case of moral insanity where any strong motive, or passion, or other exciting or adequate motive is found in the evidence. Hence, where the criminal act can be traced to a desire of gain, or to hatred, revenge, jealousy, or any other strong passion, excited by drunkenness, the act must be ascribed to such motive or impulse, and not to that irresistable impulse which is said to constitute the distinguishing characteristic of the disease."

Truly unfortunate has it been for our professional specialty, that the term "moral insanity" has ever had mention. The phrase itself is a luckless invention, not only liable to an infinitude of misconception, but conveying ideas calculated wholly to mislead. It is as if there was some separate kind of insanity, located in some *terra incognita* which no man has yet discovered, wholly independent of the brain or any of its functions or operations. What is its seat or what are the organs of its abode or production, are questions which those who employ the term are themselves puzzled to answer. It does not seem to be considered by those who give currency to the expression that the whole idea implies another centre of sensations, emotions, or passions, than their legitimate one, the brain.

In the first place, it may seriously be questioned whether such a case as is usually described to set forth the idea, is ever actually seen. Experience brings before the mind a multitude of cases, not actually realizing the full idea, but which are close approximations to it. Now it is this close resemblance between cases which do exist and a certain ideal of disease borne in the imagination which leads us astray. The small difference which does exist between the case which every one has in hand and the ideal one, is always enough to destroy the value of the instance.

It has always seemed as if all that is included in the idea of moral insanity, might be better disposed of by a closer reference to phenomena of insanity which are of every day occurrence. Every one realizes how few of the delusions of the insane mind are ever revealed, and how readily they are revealed under one set of circumstances and concealed under others. All insane asylums abound in cases of unquestionable mental disease, where its palpable manifestations are so slight that the unskilled observer would doubt its existence. A certain suspicious reserve, a mysterious shyness of manner, some haughtiness of bearing, or some thing marked and singular in gait, or tone of voice, some strange attachment to a particular seat, or special stress applied to the doing of some trivial act, may be all that distinguishes the individual from other men. Yet one guided by experience has no hesitation in declaring such cases to be instances of latent delusion; and is prepared for the sudden exhibition of extreme or violent acts of which any of these almost unobserved antecedent peculiarities furnish the explanatory key. In such cases, the extent of the disease is not at all measured by what appears on the surface.

The delusion which has possession of the mind may even have no outward form of manifestation whatever, that can be detected, and yet may give rise to all those singular, inexplicable, and perhaps violent acts, which a failure to explain by any anterior indications of delusion has styled moral insanity. It is very easy, especially with those much conversant with the insane, to conceive a case possessing all the attributes assigned to the form of disease here called in question; but before admitting any such case as an existing fact, the possibility of a latent delusion underlying its characteristic perversities of conduct should be deeply considered.

It may be said, in reply to this view of the subject, that it assigns to delusion too indispensable a place in all cases of insanity, whereas it is well known that in many cases of even partial mania no such feature is believed to exist. This does not necessarily follow. Delusion among the insane may be supposed to bear about the same relative part in their unnatural acts that a well defined motive does in the acts of those who reason correctly. Persons possessed of reason perform the larger portion of their acts from no actually considered motive of which they are conscious. Acts are done from an impulse which is, after all, the result of some former reasoning process. So the phenomena of moral insanity, so called, may follow some former delusive process of thought of which the individual himself has no consciousness, and which, of course, no skill of another can detect. If this explanation is not in all cases satisfactory, it at least has the merit of enabling us to pass a stumbling-block now almost invariably thrown in our way whenever we appear in court.

THE PEOPLE'S INSTRUCTIONS.

"In applying the principles of the law of insanity as thus defined, to the particular circumstances of this case, the Court instructs the jury on the part of the People, and in their behalf, that if they believe from the evidence:

"*First.*—That the mind of the accused was affected with insanity, only while in a state of drunkenness, and that with a knowledge of this predisposition and of right and wrong, the accused voluntarily put himself in that state and committed the act with which he is charged, the act in that case is criminal in the same degree as if there had been no predisposition to insanity when under the influence of drunkenness.

"*Second.*—That even though the jury should find that the accused was affected with insanity by reason of a delusion in regard to his wife's fidelity, yet if they further find that at the time he committed the act he had a perfect knowledge of right and wrong with respect to the act itself, and was under no delusion with respect to its moral quality, then the law regards him as a moral agent in the commission of the crime and subject to its penalty.

"*Third.*—That insanity produced immediately by intoxication does not destroy responsibility, and if the jury find from the evidence that the accused, while sane and responsible, voluntarily intoxicated himself, and in that state committed the act, they will find him guilty.

"*Fourth.*—That if the jury believe from the evidence that the accused, when free from the influence of intoxicating drinks was uniformly sane and rational, and forbore all violence towards his wife, and that for a series of years prior to the commission of the act in question, he was accustomed, in fits of intoxication, to use violence upon her, and knew that such violence was the immediate result of such intoxication, and that having such knowledge he voluntarily made himself intoxicated on the day of the homicide charged in the indictment, and that such act was the immediate result of such intoxication, then the defendent is responsible for the crime, although he might have been laboring under some insane delusion at the time.

"*Fifth.*—That if the act was done by the accused under the influence of passions excited by drunkenness, or jealousy, or hatred, without provocation on the part of the deceased, or any danger to life or limb, that in that case the accused is not entitled to be excused from the consequences of the act on the ground of moral insanity, however strong or irresistable the passion may have been under which the act was perpetrated.

"*Sixth.*—That if the jury find that the accused was actuated by malice, jealousy, or other feeling of hatred, or from passions excited by drunkenness, at the time of the killing, then he is guilty of the crime of murder, though the jury may find that he was affected with insane delusion with respect to his wife's chastity."

Now, this will certainly be regarded, in view of some points in the evidence, as rather hard measure for the prisoner. The second and fourth parts of the instructions must bear upon the accused with little less than fatal effect. Granting the great material fact that the prisoner is an insane man, it hangs his only hope upon what a jury may conceive to be a "perfect knowledge of right and wrong with respect to the act itself." The effect of this position is to show that a mind may be radically diseased, and yet, upon the very point on which it is diseased, a nice and logical reasoning may, and indeed does, go on as to the quality of the act being done. It forces the prisoner to become a casuist while pressing forward to a violent act, under the irresistable control of an insane delusion. If Hopp believed, on grounds insanely wrong, that his wife was wickedly unfaithful—bringing ruin and perdition on herself, and disgrace on her family—and regarded her death as necessary, and, as he informs the by-standers after the fatal blow had been struck, "meditated for ten years," could he have had "a perfect knowledge of right and wrong with respect to the act itself," as we understand the general ability of an insane mind to compass such knowledge?

The effect of setting aside the actual degree of the mental disease as a measurement of criminal responsibility, and substituting a fancied perverted use of the canons of good logic, as applied to some unnatural transaction, is seen at once. The fatal tendency of allowing a certain knowledge of right and wrong in regard to the acts of the accused to set aside any extent of insanity without that knowledge, is clearly shown in this case. When the delusion was lifted from his mind, by the absence of the object of it, as an inducement to procure her return, he actually acknowledges the wrong of his ill-treatment, attributes it to liquor, and promises, under oath, to drink no more. Yet who does not see how unjust to the prisoner is this self-conception of his wrong when it is viewed by others in connection with his disease? In the "good time coming" we shall probably have done with all this, and deal more with the simple question of the actuality and degree of the insanity, and of the disjointing of the reasoning processes generally.

INSTRUCTIONS FOR THE DEFENSE.

"And the Court, on the part and behalf of the accused, further instructs the jury:—

"*First.*—That if they believe from the evidence that the accused was at the time of the killing not drunk, but laboring under a fixed and insane delusion as to his wife's infidelity and want of virtue, and that such delusion operated so powerfully upon his understanding and will as to render him incapable of perceiving or being sensible of the moral quality of the act, or knowing and acting upon the principle of right and wrong, in relation to the act, then such insanity entitles him to an acquittal on the ground that he was not a free moral agent.

"*Second.*—That if they believe from the evidence that the act of killing was the offspring and consequence of insanity in the accused, and not induced by drunkenness, hatred or malice, and that such insanity was the offspring of delusion in regard to his wife's chastity, and so great as to overcome the will and obliterate all consciousness of right and wrong with respect to the act, or induced a fixed and insane belief that its commission was one of duty, then the jury should acquit, although they may believe that the accused was capable of reasoning correctly, and impressed with clear perceptions of right and wrong, with respect to the act of killing in general.

"*Third.*—That if they believe from the evidence that at the time of the commission of the act charged, the mind of the accused was laboring under an insane delusion caused by disease and not excited by drunkenness, with respect to the existence of facts, which if true would excuse homicide—as that a known felony was about to be committed—and that overcome and impelled by such delusion the accused took the life of the deceased to prevent, in his insane belief the commission of the felony, then the act of killing must be considered the direct effect of disease and not of a mind capable of volition or choice.

"*Fourth.*—That if they believe from the evidence, that the homicide committed by the prisoner was not the act of a man operated upon by motives and governed by the will, but the result of a mere uncontrollable impulse, communicated to his mind from insanity of the moral powers, and not by motives of hatred, jealousy, or drunkenesss, or other passion impelling to the act, then the act was one of moral insanity. But in determining this question, the jury should have reference to the more exact definition of moral insanity given in previous instructions on this subject.

"*Fifth.*—That if they find from the evidence that at the time of the killing, the mind of the accused was affected with insanity caused by disease, and that the act was the effect of such insanity and not of passions or insane delusions resulting direct from voluntary drunkenness, then the defendent stands excused on the ground of insanity. But in such case the jury must be satisfied that the insanity was of such a nature as to obscure the mind with respect to the moral quality of the act or induce the belief that it was necessary in self-defence; for though insane delusion may have existed, yet if it was not of such a character as will excuse homicide, the accused is not entitled to an acquittal on that ground.

"*Sixth.*—That if they find that at the time of the homicide the accused was affected with such insanity as would excuse from the consequences of acts otherwise criminal, then the homicide is excusable on the ground of insanity, though the jury may believe from the evidence that such insanity was occasioned by past excesses of drunkenness. Where a person is insane he is not responsible criminally, although such insanity be remotely caused by indulgence in spirituous liquors. But it is otherwise if he is intoxicated at the time, and his insanity or delirium is the direct and immediate effect of such intoxication.

"*Seventh.*—That if the jury are convinced from the evidence, that the killing was the immediate effect of an insane delusion, concerning his wife's chastity, so affecting his mind as to control the will and obscure his perception of right and wrong with respect to the act, and that such state of mind was not the effect of passions excited by ardent spirits, then the act is excusable on the ground of insanity, though he may have been drinking. But the conviction of the mind on this point should be clear, and care should be taken not to confound passions excited by liquor with those which are the natural effects of insanity. For if insanity existed, but would not have manifested itself in homicide if it had not been stimulated by excitements caused by liquor, then the act is not excusable on the ground of insanity. But if the jury can reconcile the evidence tending to prove drunkenness, with a conviction drawn from the evidence that the act was one of insanity and not the effect of drunkenness, it is their duty to refer the act to insanity, and acquit the prisoner on that ground.

"*Eighth.*—That if the jury shall find that the accused, before the commission of the act, was affected with insanity of a nature to obscure and overcome his moral perceptions with respect to the act committed, then the burden of proof is upon the prosecution to show that he was not affected with such insanity at the time of the killing."

An examination of the above will show how little the accused has to hope from any instructions which will not recognize the disease and the vicious habit as two incidents, to be separately considered. The first section, for instance, can be of no effect, because the defence does not deny the fact of the drinking on the day of the homicide, probably to the extent even of intoxication. The insanity and the drunkenness are put too much in the light of incompatable states to enable the idea of the former much to aid the accused. The fatal idea that the prisoner was *either* insane or drunk, was that which a juryman, not much in the habit of thinking, would most likely entertain; and the instructions of the Court fail to give the prisoner all the advantage which his defence claimed for him in not recognizing drunkenness as possible to be superadded to insanity, and allowing the onus of the crime to fall upon the permanent state, and not upon the accidental one. The references to the condition of drunkenness through the following sections of the chapter, except the last, sustain the same connection of the two ideas, and allow the mind the easy duty of merely holding the two states as incompatible—connecting the one always with the idea of guilt, and the other only with the possible one of innocence. If the idea of the eighth section had been the leading one through all the instructions to the jury, it is evident that a new complexion would have been given to the case.

It is an unfortunate omission in these instructions that the minds of the jury were not as much carried back to the idea of premeditation as the evidence warranted, but allowed to contemplate the act as one of impulse merely. In what does the actual guilt of the crime of murder consist? Not alone, or principally even, in striking the blow that deprives of life, but in that premeditation which resolves on, and shapes the manner of the deed. The law recognizes this by holding him guilty who aids or countenances this premeditation of a crime. Now, taking the prisoner's solemn declaration, an hour after this homicide, it had been the intention for years. That deliberate purpose could not have been the effect of drunkenness; and if not, what was it but insanity?

### CONCLUDING INSTRUCTIONS.

"In conclusion, the Court instructs the jury, that it is their duty to give a careful consideration to all the facts and opinions in proof, throwing light upon the insanity of the prisoner at the time in question. On this subject, medical opinions and evidences are entitled to attentive and respectful consideration. And if the act is proved to the satisfaction of the jury, by the weight and preponderance of the evidence, to have been one of insanity only, the prisoner is entitled to an acquittal, *though that defence should not be proven beyond all reasonable doubt.*

Whatever of criticism may have been bestowed on any of these preceding observations, the italicised portion of the above is a concession to the plea of insanity that will certainly procure for Judge Manierre the regard of those entrusted with the interests of the insane. It is the first time, to our knowledge, that insanity has been allowed the same privilege as actual crime, in having the "benefit of a doubt." Hitherto, while all doubt in ordinary criminal prosecutions enured to the prisoner's benefit, doubts in regard to sanity did those who denied it in plea, no good whatever. The proof of insanity must be positive, or else was set aside as of no sort of weight. Slight though this enunciation may be, it should be treasured as the dawn of new and better things in this department of jurisprudence.

The prisoner was convicted of murder, though it is believed that another trial may be had.

